# Are future physicians practicing safe caffeine consumption? A structured narrative review of caffeine and energy drink use among medical students

**DOI:** 10.3389/fpubh.2026.1935826

**Published:** 2026-09-22

**Authors:** Adan Ahmed, Carolyne Maina, Arsalan Akhtar

**Affiliations:** School of Medicine, St. George's University, Saint George's, Grenada

**Keywords:** academic stress, caffeine, energy drinks, health behavior, medical students, professional identity, sleep deprivation

## Abstract

**Background:**

Caffeine reaches medical students through coffee, tea, soft drinks, tablets and energy drinks, but these products differ in composition and exposure. Reported prevalence varies because studies use different definitions, recall periods and samples.

**Objective:**

To synthesize evidence on caffeine and energy drink use among medical students, distinguish product-specific from total-caffeine exposure, examine associated factors and outcomes, and appraise evidence quality.

**Methods:**

This structured narrative review searched PubMed, Scopus and Web of Science, supplemented by Google Scholar and citation searching, for peer-reviewed English-language empirical studies published from 1 January 2000 to 30 June 2026. The final search was run on 30 June 2026; the full search strategies and the study selection flow are reported in the main text. SANRA and applicable PRISMA 2020 reporting items guided conduct. Cross-sectional studies were appraised using the eight-domain Joanna Briggs Institute checklist. Heterogeneous exposure definitions and recall periods precluded pooling.

**Results:**

Fourteen studies involving 5,815 participants met the criteria: 13 cross-sectional surveys and one repeated cross-sectional mixed-methods study. Energy drink estimates ranged from 41.4% past-six-month use in Kazakhstan and 50.0% ever use in Jordan to 62.0% current use in India, 63.9% reported use in Pakistan and 75.8% use among Polish medical students. Total-caffeine studies reported 93.6% use in South Africa and 81.3% in Saudi Arabia; 52% of Saudi users consumed at least 400 mg/day. Students commonly cited wakefulness, concentration and fatigue management, particularly during examinations. Sleep was the most consistent health-related domain. No study demonstrated academic benefit. Recurrent limitations included convenience sampling, self-reported intake, limited confounder control and absent temporal ordering.

**Conclusion:**

Caffeine and energy drink use are common, but prevalence depends strongly on product definition and recall period. Evidence supports concern about high-dose or poorly timed exposure and sleep-related associations, but not causal claims about burnout, academic performance or professional impairment. Standardized measures, longitudinal studies and evaluated interventions are needed.

## 1 Introduction

Caffeine is a psychoactive stimulant delivered through products that differ materially in composition and pattern of use. Coffee and tea carry caffeine within complex beverages taken in moderate servings across the day; soft drinks and tablets add further caffeine; energy drinks combine caffeine with sugar or sweeteners, taurine, guarana and B vitamins and are often consumed rapidly and episodically (1). Evidence for one exposure cannot be transferred to another, and where a study reports only total caffeine the energy drink contribution is usually indeterminable. This review therefore treats three exposures separately: caffeinated energy drinks; coffee and other traditional caffeinated beverages; and total daily caffeine from all sources.

Global energy drink use is substantial. A meta-analysis of 192 studies (*n* = 1,120,613) estimated worldwide lifetime prevalence at 54.7% (95% CI 48.8–60.6) and daily use at 8.8% (95% CI 6.3–11.4) (2), while a pooled estimate confined to university students was 42.9% across 71 studies (3). Neither figure is specific to medical students. Energy drink use has been associated with smoking, heavy drinking and higher BMI (39), and with anxiety and mood symptoms at higher intakes (4, 5). By contrast, an umbrella review of 201 meta-analyses of observational and 17 of interventional research reported that moderate coffee intake was more often associated with benefit than harm (6). That contrast concerns coffee, is largely observational, and does not license inference about high-dose or rapidly consumed energy drinks.

The European Food Safety Authority (EFSA) regards single doses up to 200 mg and habitual intake up to 400 mg/day as safe for healthy non-pregnant adults, but warns that 100 mg near bedtime may disturb sleep (7). FDA identifies 400 mg/day as generally unassociated with adverse effects (36), while WHO guidance treats energy drinks as distinct health and regulatory concerns (8). Students often use caffeine at night. Examinations, early clinical schedules, fatigue and stress encourage stimulant use during medical training (9, 38). Caffeine may improve vigilance and memory (10–12), but does not establish better grades, clinical reasoning or wellbeing. Medical students will counsel patients about sleep and stimulant risks (13, 14). Prevalence estimates remain incomparable because studies measure different exposures, periods and outcomes in convenience samples.

This structured narrative review therefore aimed to: (1) identify and critically appraise primary empirical studies of caffeine or energy drink use among medical students and in the transition to junior practice; (2) synthesize prevalence and use patterns by exposure without combining non-equivalent measures; (3) examine academic, psychosocial, health and professional associations; (4) compare observed intakes with international reference values; and (5) set out measurement, design and intervention priorities for future work.

## 2 Methods

### 2.1 Review design and reporting framework

This study used a narrative review design to critically synthesize the existing evidence on caffeinated energy drink consumption among medical students. A structured narrative approach was used because the literature is heterogeneous in exposure definition, recall period, sampling and outcome measurement, so a pooled prevalence estimate would have no coherent referent and the resulting heterogeneity would not be interpretable. Methodological transparency was maintained through predefined eligibility criteria, documented screening, a flow diagram and formal critical appraisal. The review followed the SANRA criteria for narrative review quality (15) and adopts the PRISMA 2020 reporting elements that apply to non-pooled syntheses: a specified search strategy, pre-defined eligibility criteria, documented screening, a flow diagram and formal critical appraisal (16).

### 2.2 Information sources and search strategy

A structured literature search was conducted using PubMed, Scopus, Web of Science, and Google Scholar. Studies published in English between January 2000 and June 2026 were searched using combinations of keywords including energy drinks, caffeinated beverages, caffeine consumption, medical students, physician trainees, academic performance, sleep, stress, and mental health. Reference lists of relevant articles were also screened to identify additional eligible studies. Search concepts combined caffeine source (energy drink, caffeine, coffee, caffeinated beverage), population (medical student, medical undergraduate, physician trainee, junior doctor) and outcome (prevalence, consumption, sleep, stress, mental health, academic performance, burnout), using database-specific controlled vocabulary and field tags. Complete search strings as executed are reported in Appendix A.

### 2.3 Eligibility criteria and study selection

The review included peer-reviewed studies of caffeine or energy drink use among undergraduate or postgraduate medical students if they reported prevalence, consumption patterns, determinants, health outcomes, or academic and professional implications. Studies of general university populations were eligible only when they presented medical student data separately. We excluded editorials, conference abstracts, commentaries, non-English publications and studies that did not define the exposure measured. Junior doctors were included only where researchers explicitly examined continuity from medical school. To prevent unlike evidence from being combined, we separated sources into two categories. Primary evidence comprised empirical studies reporting original data from the target population; only these studies appear in Table 1 and inform claims about prevalence and determinants. Reviews, commentaries and pharmacological studies were retained only as contextual sources and are identified accordingly in the text. Titles and abstracts were screened against the predefined criteria, followed by full-text assessment. Two authors (A.A. and C.M.) screened independently at every stage, with disagreements resolved by the third author (A.K.). Reasons for full-text exclusion were recorded for the flow diagram (Figure 1). Where a published sample size was ambiguous, or where an abstract and a results section disagreed, the full text and primary data tables were consulted and the discrepancy recorded.

**Figure 1:**
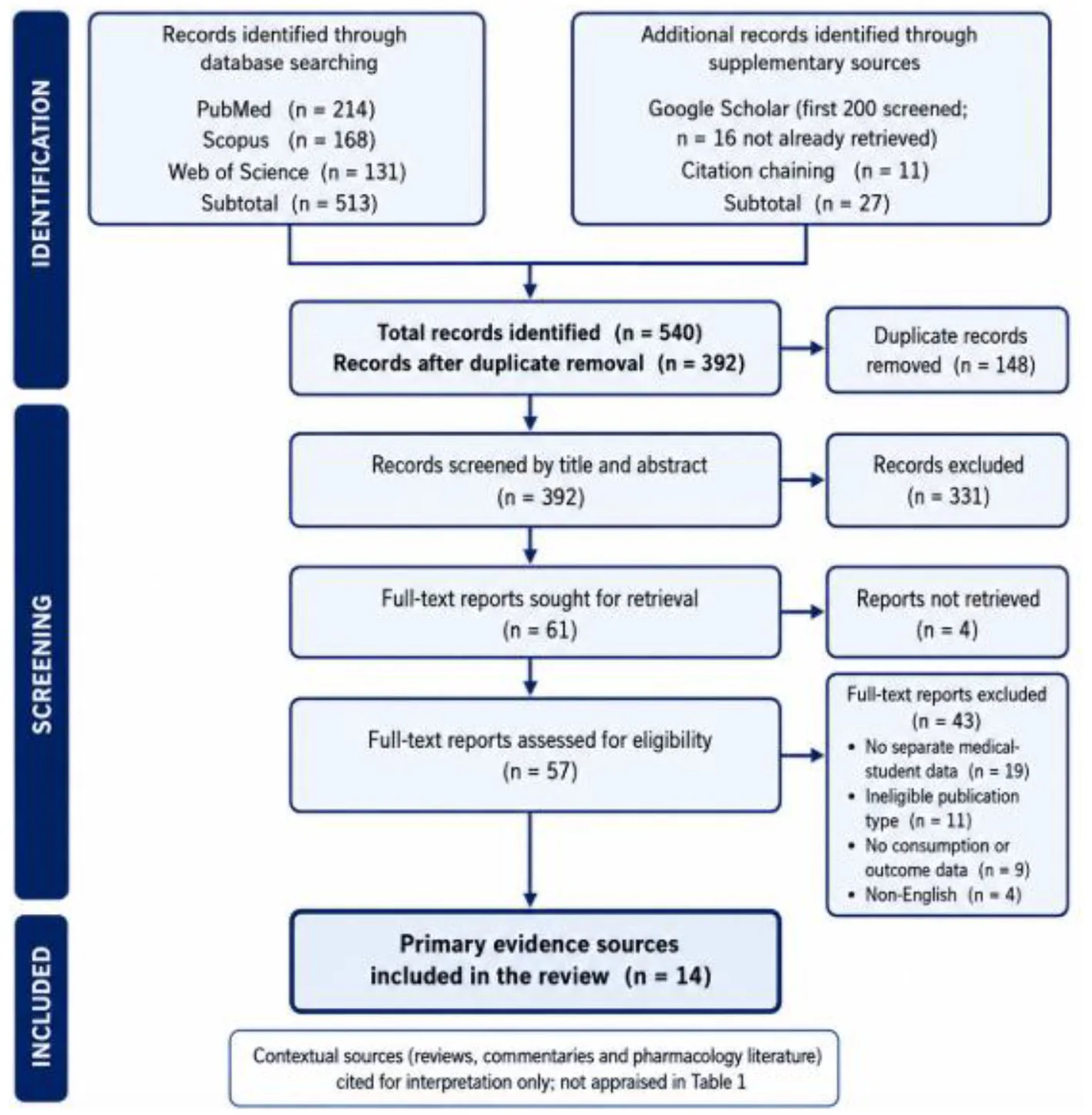
Study identification and selection flow diagram. The diagram shows the records identified through database and supplementary searches, duplicate removal, title and abstract screening, full-text assessment, reasons for exclusion, and final inclusion of 14 primary studies.

**Table 1:** Characteristics and methodological appraisal of the 14 primary studies.

Study and setting	Design and sample	Exposure measured	Principal finding	JBI	Appraisal
Lee et al. (21), South Africa	Cross-sectional; *n* = 360 of 389 invited (90.5%)	Total caffeine, all sources	93.6% reported caffeine use; 62.6% cited academic purposes; coffee was the commonest source (88.2%)	5/8	Moderate: single institution, self-report
Arif et al. (35), Pakistan	Cross-sectional; *n* = 104 fourth-year students	Energy drinks	Prevalence not reliably reported: the abstract and the results conflict (Section 3.2.1), so the estimate is excluded. Among users, 45.2% consumed less than monthly and 5.8% daily	3/8	High: one class, inconsistent prevalence reporting
Babar et al. (24), Pakistan	Cross-sectional; *n* = 332	Energy drinks	212 of 332 (63.9%) reported ever using energy drinks; alertness was the commonest perceived benefit; occasional use predominated	5/8	Moderate: single site, confounding unresolved
Dahlawi et al. (13), Saudi Arabia	Cross-sectional; *n* = 593	Caffeinated beverages	Coffee commonest and energy drinks least common; no association detected between caffeinated beverage consumption and academic performance	6/8	Moderate: self-reported intake and GPA
Mahmood et al. (30), Iraq	Cross-sectional; *n* = 438, aged 18–25	Energy drinks	Use was common; self-reported cardiovascular and psychobehavioural symptom measures differed by use pattern	6/8	Moderate: single university, residual confounding
Mussabekova and Tukinova (19), Kazakhstan	Web-based cross-sectional; *n* = 764	Energy drinks	41.4% reported use in the preceding 6 months; 16.2% reported current use	5/8	Moderate: online self-selection; recall period drives the estimate
Qasem et al. (25), Jordan	Cross-sectional; *n* = 307, six public universities	Energy drinks	50.0% reported ever use; regular use varied with region, living alone, alcohol use and daily coffee (all P < 0.05); risk awareness did not track lower use	7/8	Lower to moderate: strongest sampling frame
Pulla et al. (14), India	Cross-sectional; *n* = 560, all years	Total caffeine, all sources	Mean reported intake 151.3 mg/day; 38.1% reported more than 300 mg/day during examinations; coffee the principal source	6/8	Moderate: questionnaire dose estimation, single city
El Seifi et al. (22), Saudi Arabia	Cross-sectional; *n* = 305, stratified random sampling	Total caffeine, all sources	81.3% reported caffeine use; 52% of users reported at least 400 mg/day; intake was inversely correlated with WHO-5 wellbeing	7/8	Lower to moderate: stronger sampling; directionality unresolved
Hossain et al. (18), India	Cross-sectional; *n* = 226, simple random sample	Energy drinks	62.0% reported current use; 75% of users consumed less than one drink per week	6/8	Moderate: single college, limited confounder adjustment
Mularczyk-Tomczewska et al. (20), Poland	Cross-sectional; *n* = 871 (367 medical, 504 non-medical)	Energy drinks	75.8% of medical and 73.2% of non-medical students reported use, not significantly different despite higher risk awareness among medical students	6/8	Lower to moderate: informative comparison group; self-selection remains
Phung et al. (23), United States	Cross-sectional online survey; *n* = 121, years 1–3	Total caffeine, all sources	Mean intake 114.3 mg/day (SD 625.7); the rise across years 1–3 was not significant (P = 0.09). Coffee led (96/121), then tea (85/121), soft drinks (51/121), energy drinks (33/121)	3/8	High: small sample, SD exceeds mean fivefold, unvalidated screening items
Singla et al. (27), Pakistan	Cross-sectional; *n* = 300, aged 18–30	Total caffeine; intake and timing	Higher intake was associated with poorer sleep quality on the Pittsburgh Sleep Quality Index, particularly with late-day consumption; findings for restless-legs symptoms were less consistent	6/8	Moderate: validated sleep instrument
Vella-Fondacaro et al. (26), Malta	Repeated cross-sectional mixed methods, 2015 and 2024; *n* = 534 (361 students, 173 junior doctors)	Energy drinks and coffee, reported separately	61–66% lifetime use; in 2024 both energy drink and coffee use rose during examinations; the 2015 sex gap was no longer significant in 2024; use continued among junior doctors	7/8	Lower to moderate: strongest design; no individual follow-up

JBI = number of the eight Joanna Briggs Institute checklist domains satisfied. Overall rating: lower to moderate concern, 7–8 domains; moderate, 5–6; high, 4 or fewer. “Lower to moderate” denotes comparatively stronger methods within an evidence base that remains observational and self-reported throughout.

### 2.4 Data extraction, appraisal and synthesis

A structured extraction form captured country, setting, design, sampling, sample size, response rate, participant stage, exposure definition, recall period, caffeine source, outcome measures, statistical approach, principal findings and limitations. Extraction was performed by one author and checked in full by a second. Cross-sectional studies were appraised using the eight-domain Joanna Briggs Institute checklist for analytical cross-sectional studies, covering inclusion criteria, description of subjects and setting, validity of exposure measurement, objective outcome criteria, identification of confounders, strategies to address confounding, validity of outcome measurement and appropriateness of analysis (17). Two authors appraised each study independently and resolved discrepancies by discussion. The number of domains satisfied is reported in Table 1 alongside a summary judgement; the checklist was applied with minor adaptation to the repeated cross-sectional study, for which comparability of the two waves was considered additionally. No study was excluded on the basis of its appraisal; the rating is used instead to weight the confidence attached to each finding.

Since exposure definitions and recall periods were not equivalent, statistical pooling was not undertaken. Findings were synthesized by exposure first, energy drinks separately from total caffeine, then by prevalence, determinants, health associations and academic or professional implications. Where the literature converges we identify what makes the convergence credible; where it diverges we attribute the divergence to a methodological source before considering substantive explanations. Given that all primary studies but one are cross-sectional, associations are reported as associations throughout, and directional language is used only where a claim is flagged as a hypothesis requiring prospective testing.

## 3 Results

### 3.1 Study characteristics and appraisal of the evidence base

Fourteen primary empirical studies published between 2009 and 2026 met the eligibility criteria, with a combined 5,815 participants: 13 cross-sectional surveys and one repeated cross-sectional mixed-methods study. Settings were South Africa, Pakistan, Saudi Arabia, Iraq, Kazakhstan, Jordan, India, Poland, Malta and the United States. Evidence remains sparse for sub-Saharan Africa beyond a single 2009 study and absent for Latin America, East Asia and Oceania, which is a geographic gap rather than evidence of low use. Sample sizes ranged from 121 to 871; where reported, response rates ranged from 49% to 90.5%, and nine studies did not report one, which prevents any assessment of non-response bias. Only two studies sampled more than one institution. All sample sizes and exposure estimates were verified against the primary publications before synthesis. Characteristics and appraisal are given in Table 1.

The appraisal identified four recurrent weaknesses. First, sampling: twelve of fourteen studies recruited convenience samples from a single institution, so estimates describe the students who chose to respond at one school rather than a defined population. Second, exposure measurement: only four studies used a previously validated instrument, and all relied on retrospective self-report of brand, serving size and frequency, which is subject to recall error and social desirability bias in a population that knows caffeine overuse to be undesirable. Third, confounding: most analyses were bivariate, and multivariable models rarely adjusted for baseline sleep, workload, examination proximity or concurrent distress. Fourth, design: no included study can establish temporal ordering. The multi-wave Maltese study and the stratified Saudi and randomly sampled Indian studies were comparatively stronger, and are weighted accordingly in the synthesis below.

### 3.2 Exposure-specific prevalence and consumption patterns

#### 3.2.1 Energy drink use

Energy drink use was common in several samples, but no single prevalence range represents the evidence adequately, because the estimates describe different recall periods and denominators. Reported figures include 41.4% past-six-month use in Kazakhstan (*n* = 764), 50.0% ever use in Jordan (*n* = 307), 62.0% current use in India (*n* = 226), 63.9% ever use in Pakistan (*n* = 332), 61–66% lifetime use across two Maltese waves (*n* = 534) and 75.8% among Polish medical students (*n* = 367). A lifetime estimate will exceed a current-use estimate in the same population by construction, so the apparent spread is substantially an artifact of the question asked. Table 2 presents each estimate with its exposure label and the constraint that limits comparison.

**Table 2:** Energy drink estimates and the constraints on comparing them.

Reference	Sample	Measure as defined by the authors	Estimate	Interpretive constraint
Mussabekova and Tukinova (19)	Kazakhstan; *n* = 764	Past 6 months; current use	41.4%; 16.2%	Self-selected web sample; the two windows differ 2.6-fold within one study
Qasem et al. (25)	Jordan; *n* = 307, six universities	Ever use	50.0%	Multi-institution frame; ever use has no intensity threshold
Hossain et al. (18)	India; *n* = 226, random sample	Current use	62.0%	Not comparable with lifetime estimates; 75% of users consumed less than weekly
Babar et al. (24)	Pakistan; *n* = 332	Ever use	63.9% (212/332)	Single institution; investigator-designed items
Vella-Fondacaro et al. (26)	Malta; *n* = 534, two waves	Lifetime use, 2015 and 2024	61–66%	Only estimate using one instrument at two time points; separate samples, so secular change and cohort composition cannot be separated
Mularczyk-Tomczewska et al. (20)	Poland; *n* = 367 medical of 871	Any use, at least occasionally	75.8%	Highest figure but the most permissive threshold; the matched non-medical figure was 73.2%, not significantly different
Arif et al. (35)	Pakistan; *n* = 104	Ever use/never use	78.8%	Single-institution sample; ever use does not indicate current consumption, frequency or dose.

Frequency data further qualify the headline percentages. In the Indian sample, 75% of energy drink users reported occasional use of less than one drink per week (18); in Kazakhstan the current-use estimate of 16.2% was well below the past-six-month estimate of 41.4% (19); in Poland daily or near-daily intake was reported by 9.2% of respondents against 75.8% of medical students reporting any use (20). Prevalence and intensity are therefore different quantities, and a headline figure conceals the distribution entirely. Frequency and dose are the more policy-relevant metrics.

#### 3.2.2 Total caffeine and source patterns

Studies measuring caffeine from all sources answer a different question from energy drink surveys, and their figures are correspondingly higher. In South Africa, 93.6% reported caffeine use and 62.6% identified academic purposes, with coffee the commonest source at 88.2% (21). In Saudi Arabia, 81.3% reported caffeine use and 52% of users reported at least 400 mg/day (22). In India, mean reported intake was 151.3 mg/day, rising above 300 mg/day for 38.1% of students during examinations (14). In the United States, mean intake was 114.3 mg/day and coffee was the leading source for 96 of 121 respondents, with energy drinks the least common at 33 of 121 (23).

Two implications follow. Product-specific energy drink prevalence underestimates total caffeine exposure, since coffee contributed more than energy drinks in every study reporting both. Conversely, a total-caffeine estimate cannot identify whether an association is attributable to coffee, energy drinks, timing, sugar and co-ingredients, or the circumstances of use. The 81.3% caffeine-use figure from Saudi Arabia is therefore not comparable with a 50% energy drink prevalence from Jordan, and the two categories are kept separate throughout.

### 3.3 Determinants and contexts of use

Across settings, participants reported using caffeine or energy drinks to remain awake, reduce fatigue, concentrate and extend study time (18, 21, 24–26). Examination-related increases were reported in Jordan, India and the 2024 Maltese wave, and 38.1% of Indian students exceeded 300 mg/day during examinations against a mean of 151.3 mg/day overall (14). This is the most consistently replicated pattern in the literature and the only one with within-study temporal patterning, which makes it the most credible determinant finding.

The convergence should nonetheless be read carefully. It rests on self-reported motivations rather than measured determinants: students were asked why they consume and answered in terms of academic need. Such reports are informative about how students construe their behavior but are vulnerable to rationalization and do not establish that workload is the operative cause. Only two studies modeled determinants multivariably (20, 25), and neither adjusted for measured workload.

Cross-country differences may reflect genuine cultural variation, but methodological explanations are at least as plausible and have not been excluded: single-school samples produce unstable estimates, data collected near examinations capture episodic peaks, online surveys may over-represent interested users, and serving size and labeling vary by market. No study has compared regions using a common protocol, so regional variation remains a hypothesis rather than a finding.

Knowledge did not map onto behavior. Several studies reported awareness of adverse effects alongside continued use (22, 24, 25). The most informative test is the Polish comparison, in which medical students demonstrated higher risk awareness than non-medical peers within a single sampling frame yet consumed at a statistically indistinguishable rate, 75.8% against 73.2% (20). This approximates a natural experiment on the effect of health knowledge with the student environment held roughly constant, and knowledge did not differentiate behavior. It is the strongest evidence in this review against information-only interventions. It does not, however, establish that institutional conditions override individual choice; that stronger claim would require a design capable of manipulating those conditions. Table 3 sets out the recurring cross-study claims, the pattern observed for each, and the methodological explanation that has to be excluded before a substantive reading is accepted.

**Table 3:** Critical cross-study synthesis of recurring claims.

Claim	Cross-study pattern	Methodological explanation to exclude first	What the evidence supports
Prevalence varies widely between countries	Consistent	Recall period, product definition, user threshold and sampling frame all differ	Do not pool lifetime, recent, current and dose-based measures; residual cross-national variation is much narrower than raw figures suggest
Use rises around examinations	Consistent across Jordan, India, Malta	Surveys timed near examinations may capture a peak, not a change	The best-supported determinant, with within-study temporal patterning in the Maltese waves
Medical students consume more than other students	Asserted but not supported	Rests on between-study comparison rather than within-study contrast	The single within-frame comparison found no significant difference (75.8% vs 73.2%)
Consumption increases with year of study	Weakly supported	The direct test was underpowered (*n* = 121), *P* = 0.09, SD exceeding the mean	Higher use in clinical years rests on one stronger repeated cross-sectional study; not shown within individuals
Knowledge of risk reduces consumption	Contradicted	Awareness and behavior measured simultaneously by self-report	Replicated in three samples including one with a non-medical comparison group
Caffeine improves academic performance	Not supported	Experimental cognitive gains involve isolated caffeine in laboratory conditions, not energy drinks in students	The one direct test found no association with academic performance (*n* = 593)
Caffeine worsens sleep and wellbeing	Consistent association	Simultaneous measurement; distressed or sleep-deprived students may consume more	Robust and biologically plausible; direction of effect unresolved in this population
Stimulant use persists into practice	Suggestive	Two independent samples, not a followed cohort	Use continues among junior doctors; individual continuity is not demonstrated

### 3.4 Health-related associations

Sleep was the most consistent health-related domain. Higher or more poorly timed caffeine intake co-occurred with worse sleep measures among medical students and physicians, with late-day and night-time intake specifically implicated (27, 28). Experimental evidence strengthens biological plausibility: a systematic review and meta-analysis of 24 studies found that caffeine reduced total sleep time by 45 min and sleep efficiency by 7%, increased sleep-onset latency by 9 min, increased light sleep and reduced deep sleep, and estimated that a standard coffee (107 mg) should be taken at least 8.8 h before bed to avoid measurable sleep loss (29). That threshold is directly relevant to a population whose characteristic pattern is evening consumption to extend study hours. No included student study, however, measured sleep objectively, and only one used a validated sleep instrument (27), so objective measurement of the outcome this literature most implicates is absent. A recent review of the literature on caffeine as a cause of insomnia among medical students reaches the same conclusion and is cited here as a contextual rather than a primary source (40).

Wellbeing and cardiovascular findings were weaker. Higher intake was inversely correlated with WHO-5 wellbeing in Saudi Arabia (22), and self-reported cardiovascular and psychobehavioural symptoms differed by use pattern in Iraq, consistent with the recognized sympathomimetic effects of caffeine (30, 31). Because exposure and outcome were measured simultaneously in both, these associations are equally consistent with students in poorer health consuming more caffeine. In the United States survey, 63 of 121 participants endorsed at least one adapted CAGE item, most often a perceived need to cut down (42 of 121); as the instrument was adapted rather than validated for caffeine, this indicates possible concern rather than a measured prevalence of dependence (23). Evidence about coffee's long-term associations should not be used to infer energy drink safety, and acute experimental cognitive findings concern isolated caffeine at controlled doses rather than habitual consumption of multi-ingredient products (6, 10). The apparent tension in this literature between benefit and harm is partly an artifact of these design differences rather than a genuine contradiction. Table 4 summarises the health, academic and professional outcome domains, what the evidence supports in each, and the limitations that qualify it.

**Table 4:** Health, academic and professional outcomes: what the evidence supports.

Outcome domain	Synthesis	Strength and limitation
Sleep	Higher and later caffeine intake co-occurs with poorer sleep quality in medical students; experimental data show reduced total sleep time and efficiency and altered architecture	Moderate for the acute experimental effect; low for direction in students. Self-reported sleep in all but one study; no objective measurement
Mental wellbeing	Higher intake inversely correlated with WHO-5 wellbeing; associations reported with anxiety and stress	Low. Simultaneous measurement; reverse causation equally consistent; confounding by distress likely
Cardiovascular symptoms	Self-reported palpitations and related symptoms differ by use pattern; pharmacological plausibility is established	Low to moderate. One study with objective measurement in students; otherwise self-report
Dependence-like features	A majority of one small United States sample endorsed at least one adapted screening item, most often a wish to cut down	Very low. Adapted, unvalidated instrument in *n* = 121; no validated dependence measure was used
Academic performance	No included study demonstrated benefit; the one direct test found no association with academic performance	Moderate for the absence of benefit. GPA is coarse and a single null observational result is not conclusive
Professional continuity	Energy drink and coffee use continued among junior doctors in the one study spanning the transition	Low. Separate samples, no individual follow-up; no study links student consumption to clinical performance

### 3.5 Academic and professional implications

No evidence of academic gain was found, even though the academic justification offered by students was reported consistently. No included study showed that coffee or energy drinks boost grades, exam performance, clinical reasoning, or skill acquisition, despite the fact that alertness and longer study sessions were often mentioned motivations. In a direct test with 593 students, Dahlawi et al. (13) found no association between the consumption of caffeinated beverages and academic achievement. According to Phung et al. (23) and El Seifi et al. (22), the highest consumers were disproportionately those who reported the highest levels of effort, discomfort, and sleep deficit. This suggests that consumption is better interpreted as a sign of academic strain rather than a predictor of accomplishment. Reports of improvements in cognitive efficiency may indicate respite from withdrawal rather than improvement above baseline as tolerance grows (32). It is preferable to formulate professional implications as a research topic rather than an established fact. According to Vella-Fondacaro et al. (26), the Maltese study demonstrated that the use of coffee and energy drinks continued after graduation, but there is no evidence linking student consumption to clinical competence or patient care, and the study's design cannot demonstrate that the same individuals maintained a habit into practice. Although it has been discussed in commentary (38), the conflict between using stimulants during training and then counseling patients on stimulant use has not been experimentally investigated.

## 4 Discussion

### 4.1 Principal interpretation

This review found a geographically diverse but methodologically limited literature. Energy drink use and total caffeine exposure were common in many samples, particularly where ever-use was the measure, but magnitude depended on definitions and recall periods to a degree that makes any single headline prevalence misleading. The most consistent finding concerned why students said they used these products: wakefulness, concentration and fatigue management, intensifying around examinations. That convergence across very different educational systems is difficult to attribute to cultural preference alone, and is compatible with the view that features of medical training, including extended wakefulness and the normalization of fatigue, shape consumption. Consistency of association is not, however, evidence of causation, and convergence between studies sharing a design, a self-report method and a common set of *a priori* question items may partly reflect methodological rather than substantive convergence. Keeping coffee, total caffeine dose and energy drinks distinct materially changes interpretation. Coffee dominated total intake in every study reporting sources, whereas energy drink surveys captured a behavior that was frequently occasional: three-quarters of Indian users consumed less than weekly, and daily intake was under 10% in the Polish sample. A synthesis that merges the two produces conclusions that are not supported for either.

### 4.2 International guidance, sleep, burnout and health promotion

The EFSA and FDA reference value of roughly 400 mg/day is a helpful starting point, but it needs to be understood in its context. It does not mean that everyone should take 400 mg, that time is unimportant, or that coffee and energy drinks are interchangeable. In contrast, 38.1% of Indian students exceeded 300 mg/day during exams (14), and 52% of caffeine users in the Saudi sample reported at least 400 mg/day, placing a majority outside the range either authority considers unproblematic (22). Since the typical pattern is nighttime dosage to extend study hours, EFSA's conclusion that approximately 100 mg near bedtime may influence sleep is more relevant here than the daily total (7).

Sleep should therefore be central to both interpretation and intervention, and it is the domain where the evidence best supports specific advice. The finding that a standard coffee should be consumed at least 8.8 hours before bed to avoid measurable reductions in sleep time (29) is concrete, actionable and independent of the limitations of the student literature. Medical schools can reasonably teach caffeine source and dose estimation, labeling, timing relative to sleep opportunity, and the coffee–energy drink distinction without waiting for further prevalence surveys.

Burnout is a relevant context but should not be attributed to caffeine. A meta-analysis of 18 studies (*n* = 10,046) estimated burnout prevalence among medical students at 37.23%, with substantial heterogeneity (33). Burnout, sleep loss, stress, workload and caffeine use plausibly share antecedents and may influence one another, and none of the included studies can disentangle them. Confounding by indication is therefore highly likely, since the most distressed students are also the most likely to consume, and interventions targeting caffeine alone are unlikely to shift either exposure or outcome. Evaluated wellbeing approaches such as mindfulness-based stress reduction have shown measurable effects in this population and offer a more plausible route than caffeine-specific messaging (34). Active coping has likewise been associated with less burnout and greater resilience in medical students (37), which supports addressing the conditions that drive consumption rather than the consumption alone.

### 4.3 Implications for medical schools

A proportionate institutional response combines accurate individual guidance with attention to the training environment, without overstating what the evidence shows. Schools can incorporate caffeine source, dose and timing into existing sleep-health teaching; ensure confidential support for students reporting high intake or dependence-like features; review scheduling and assessment practices that unnecessarily compress sleep opportunity; and avoid institutional arrangements, including on-campus availability and sponsorship, that normalize high-dose or late-evening consumption. This review does not establish that any of these measures will reduce intake, and each should be evaluated rather than assumed. What it does support is that information alone is unlikely to suffice, since the students with the best knowledge of the risks consumed at the same rate as those without it.

### 4.4 Review and evidence limitations

This review was restricted to English-language, peer-reviewed publications and did not search dissertation, preprint or conference databases, so language and publication bias are possible and are likely to be material given that the geographic gaps identified include regions where relevant work may appear in Spanish, Portuguese, French or Mandarin. Google Scholar was used as a supplementary rather than fully reproducible source, and no protocol was registered. Narrative synthesis retains reviewer judgement despite structured eligibility and appraisal, and the appraisal reflects what source publications reported, so studies may have applied safeguards they did not describe. No formal assessment of publication bias was possible without pooling. The underlying evidence was limited by cross-sectional design, self-reported intake, single-institution convenience sampling, inconsistent recall periods and incomplete adjustment for sleep, stress and workload; nine of fourteen studies did not report a response rate; and geographic coverage was uneven. Together these preclude causal claims. The review's principal strengths are that it appraises rather than summarizes the evidence, keeps the three exposures distinct, reconciles prevalence estimates by recall period before comparing them, and documents a reporting inconsistency in a primary source that had propagated into later literature.

### 4.5 Future research priorities

First, longitudinal designs. The single most consequential gap is the absence of any study following the same individuals over time. Multicentre cohorts should recruit at matriculation and follow students into early practice, with repeated measurement of dose, product, timing, sleep, workload, stress, wellbeing and academic or clinical outcomes. This would establish temporal ordering without assuming that cross-sectional year-group differences represent individual change. Experience sampling around examinations would be a lower-cost partial substitute. Second, measurement standardization. Studies should report milligrams per day, time of last intake, product-specific frequency, serving size, lifetime versus current use, examination and non-examination periods, sampling frame and response rate. Digital diaries, brand capture and objective sleep measurement would reduce recall error, validated indicators of problematic use should replace adapted screening items, and core confounders should include baseline sleep, workload, stress and other caffeinated beverage use. A minimum reporting set of this kind would make future studies comparable and eventually poolable. Third, controlled evaluation of interventions. No included study evaluated one. Candidate strategies with a plausible mechanism include caffeine-label literacy and timing education, sleep-hygiene programmes, schedule and workload modification, stress-management programmes, and targeted support for students reporting high-dose or dependence-like use. Cluster-randomized, stepped-wedge or interrupted time-series designs at cohort or school level are appropriate, since individual randomization within a shared training environment invites contamination. Fourth, institutional policy research that tests rather than presumes the structural account. Multi-institutional mixed-methods work using a common protocol can examine how examinations, schedules, peer norms and product availability influence use across settings, and would resolve the confounded regional comparisons discussed in Section 3.3. Studies from underrepresented regions and direct medical versus non-medical comparisons are needed, with sleep and wellbeing as outcomes rather than consumption alone.

## 5 Conclusion

Caffeine and energy drink use is common among medical students and often increases during examinations, but estimates vary because studies measure different products, doses and recall periods. Energy drinks, coffee and total caffeine intake should therefore be reported separately. Students commonly cite wakefulness and concentration, yet no evidence demonstrates improved academic performance; the only direct study found no benefit. The association between higher or later intake and poorer sleep is the clearest and most actionable health finding. Risk awareness alone did not predict lower consumption, making information-only interventions unlikely to succeed. Predominantly cross-sectional evidence cannot establish that caffeine causes burnout, academic underperformance or professional impairment. Future studies should adopt standardized exposure measures, longitudinal designs and evaluated interventions.

## Appendix A. Database-specific search strategies

Search period 1 January 2000 to 30 June 2026; language English; all searches last executed 30 June 2026. Strings were adapted to the syntax and indexing of each database.

**Table A1:**

Database	Search string as executed	Records
PubMed	(“Energy Drinks”[Mesh] OR “energy drink^*^”[tiab] OR “caffeinated beverage^*^”[tiab] OR Caffeine[Mesh] OR caffeine[tiab] OR Coffee[Mesh] OR coffee[tiab]) AND (“Students, Medical”[Mesh] OR “medical student^*^”[tiab] OR “medical undergraduate^*^”[tiab] OR “physician trainee^*^”[tiab] OR “junior doctor^*^”[tiab]) AND (prevalence[tiab] OR consumption[tiab] OR sleep[tiab] OR stress[tiab] OR “mental health”[tiab] OR burnout[tiab] OR “academic performance”[tiab]). Filters: English, 2000/01/01 to 2026/06/30	214
Scopus	TITLE-ABS-KEY((“energy drink^*^” OR “caffeinated beverage^*^” OR caffeine OR coffee) AND (“medical student^*^” OR “medical undergraduate^*^” OR “physician trainee^*^” OR “junior doctor^*^”) AND (prevalence OR consumption OR sleep OR stress OR “mental health” OR burnout OR “academic performance”)) AND PUBYEAR > 1999 AND PUBYEAR < 2027 AND LANGUAGE(english) AND (LIMIT-TO(DOCTYPE,“ar”) OR LIMIT-TO(DOCTYPE,“re”))	168
Web of science core collection	TS=((“energy drink^*^” OR “caffeinated beverage^*^” OR caffeine OR coffee) AND (“medical student^*^” OR “medical undergraduate^*^” OR “physician trainee^*^” OR “junior doctor^*^”) AND (prevalence OR consumption OR sleep OR stress OR “mental health” OR burnout OR “academic performance”)). Timespan 2000–2026; language English; document types article and review	131
Google scholar (supplementary)	(“energy drinks” OR caffeine) AND “medical students” AND (prevalence OR consumption OR sleep OR stress). Not a reproducible Boolean interface; first 200 records by relevance screened	200 screened; 16 retained
Citation searching	Backward chaining of reference lists of all included full texts; forward citation search in Scopus for the five most-cited included studies	11
Total identified	513 from indexed databases plus 27 from supplementary sources; 148 duplicates removed, leaving 392 records for screening	540

Record counts correspond to the flow of studies shown in Figure 1. Google Scholar was used only as a supplementary source because it does not support reproducible Boolean queries or exportable result sets.
